# Supplementary material for: Spatial and Temporal Constraints on the Composition of Microbial Communities in Subsurface Boreholes of the Edgar Experimental Mine
Source: Microbiol Spectr. 2021 Nov 10;9(3):e00631-21. doi: 10.1128/Spectrum.00631-21 (PMC8579930; doi:10.1128/Spectrum.00631-21)
Supplement: SUPPLEMENTAL FILE 2 — Supplemental material. Download Spectrum.00631-21-s0001.pdf, PDF file, 0.7 MB [file spectrum.00631-21-s0001.pdf]

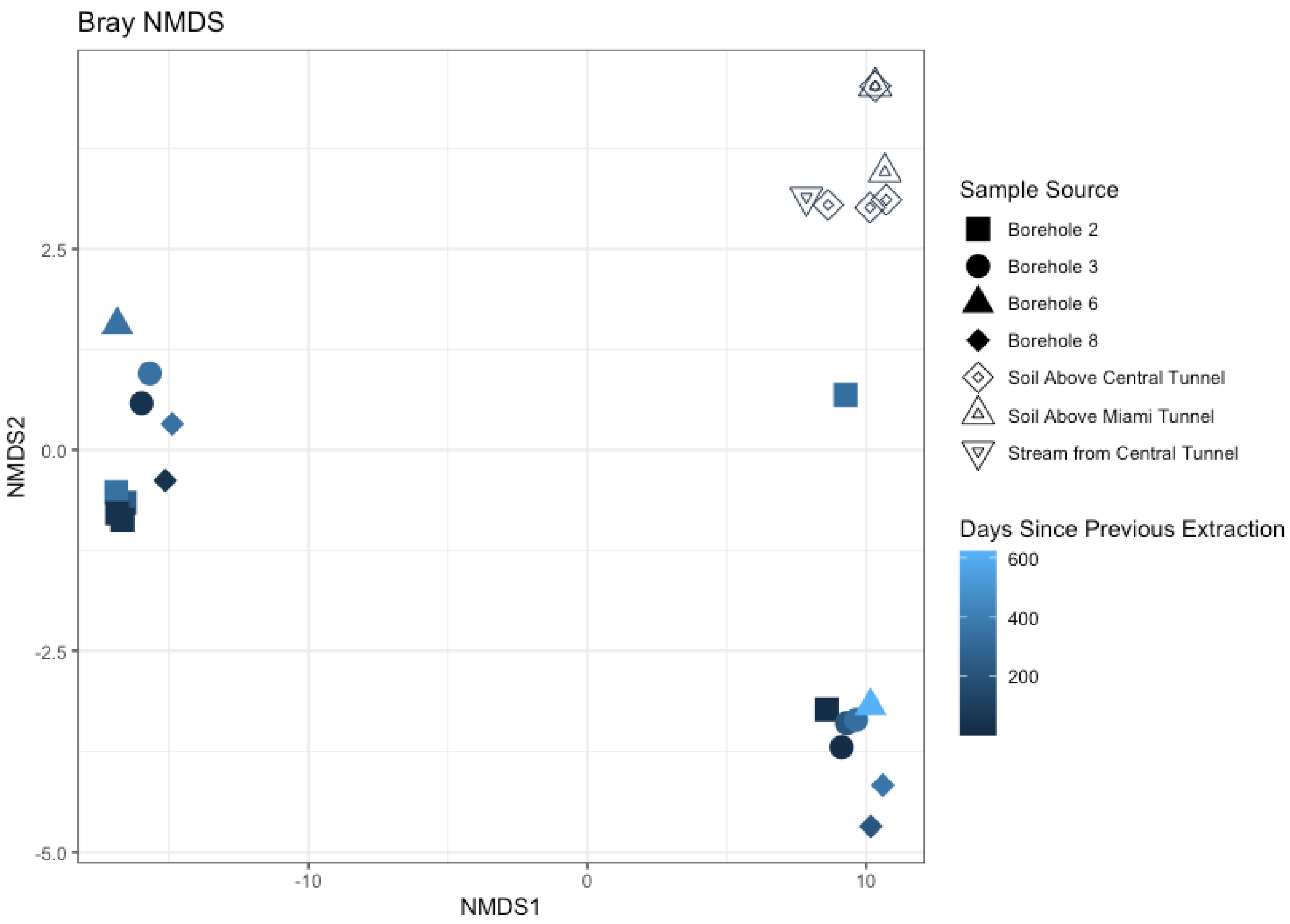

Figure S1. NMDS Plot of time since previous extraction for each sample. Clustering does not appear to show any distinct trends or associations. Soil samples are indicated as such and do not include a time variable due to only a single collection period. An Adonis test demonstrates that the time since the previous extraction for each sample is not significant (R^2 = 0.073, p = 0.05).

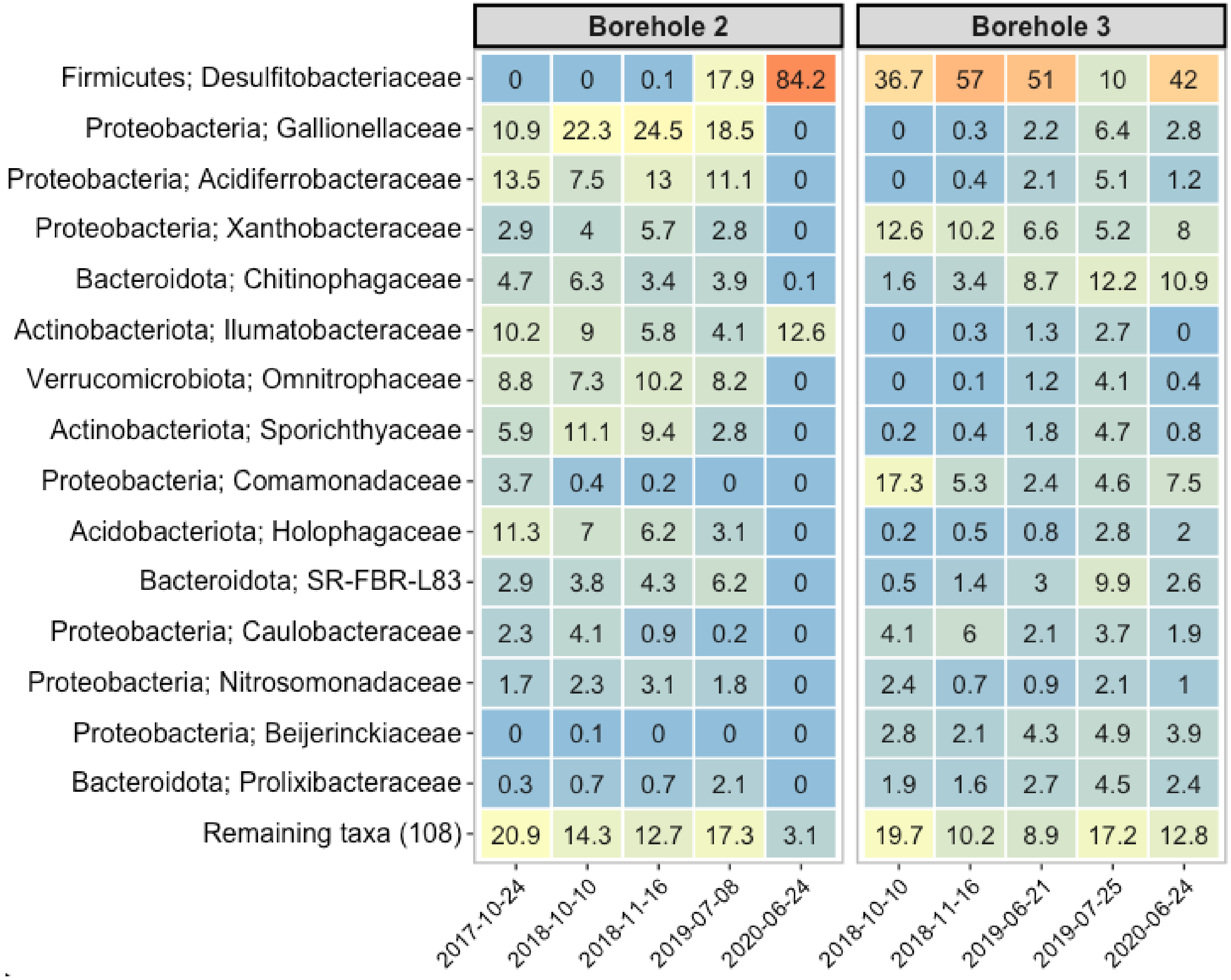

Figure S2. The top 15 family-level microorganisms within Boreholes 2 and 3 as a function time over the course over the experiment. Each column indicates the sampling date in which a sample for the respective borehole was collected. Numeric values within the box indicate the percent read abundance, also represented as a heatmap through boxes colored by the numerical ranges represented from high (orange) to low (blue). The full phylogenetic lineage (Phylum;Family) is depicted to give full coverage of the organism in abundance within each borehole or soil samples.

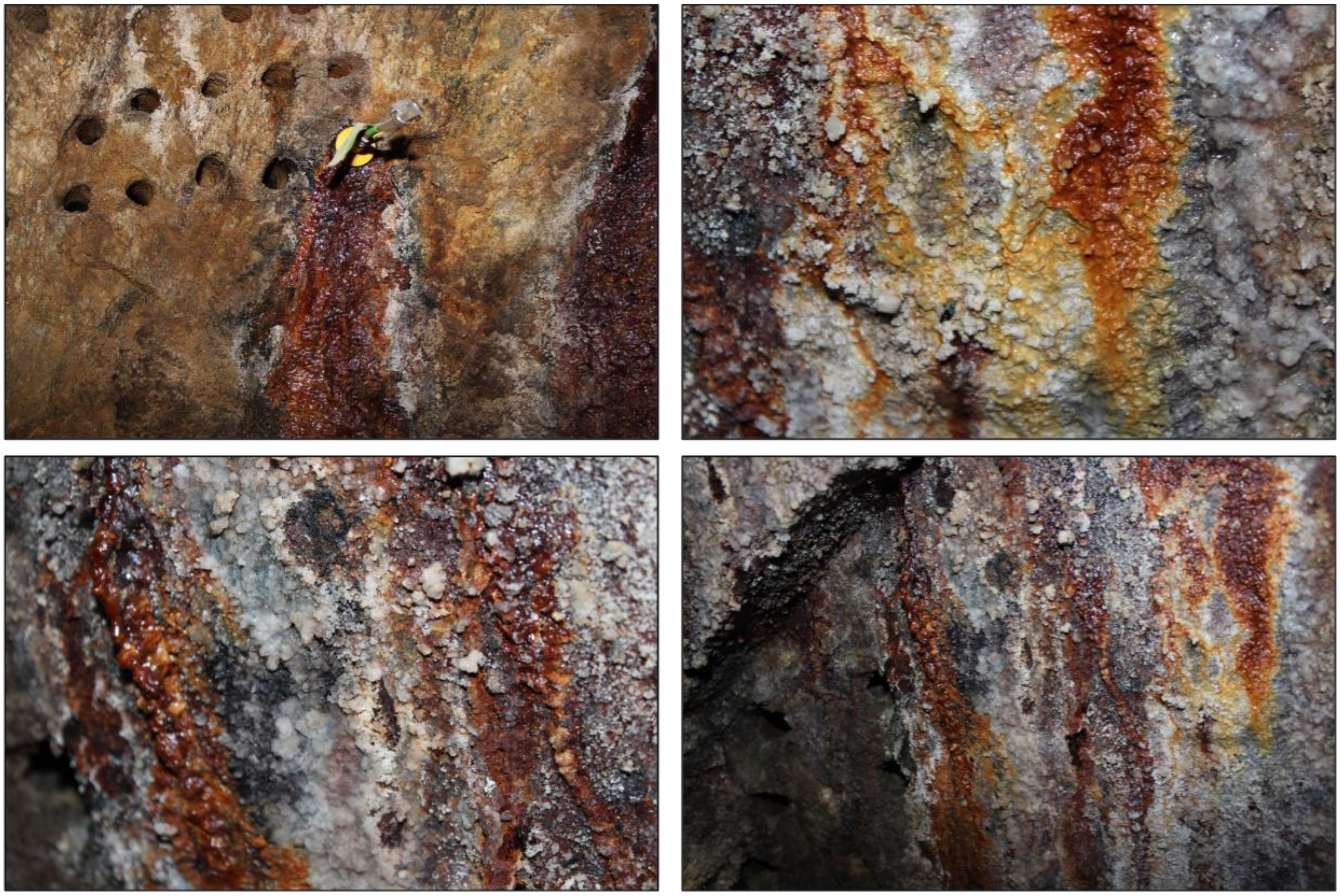

Figure S3. Biofilms collecting along the rock faces along the walls of the mine from leaking boreholes that are not packed. Discoloration from white to dark brown can be observed with crystal-like precipitates forming along the leakage path.

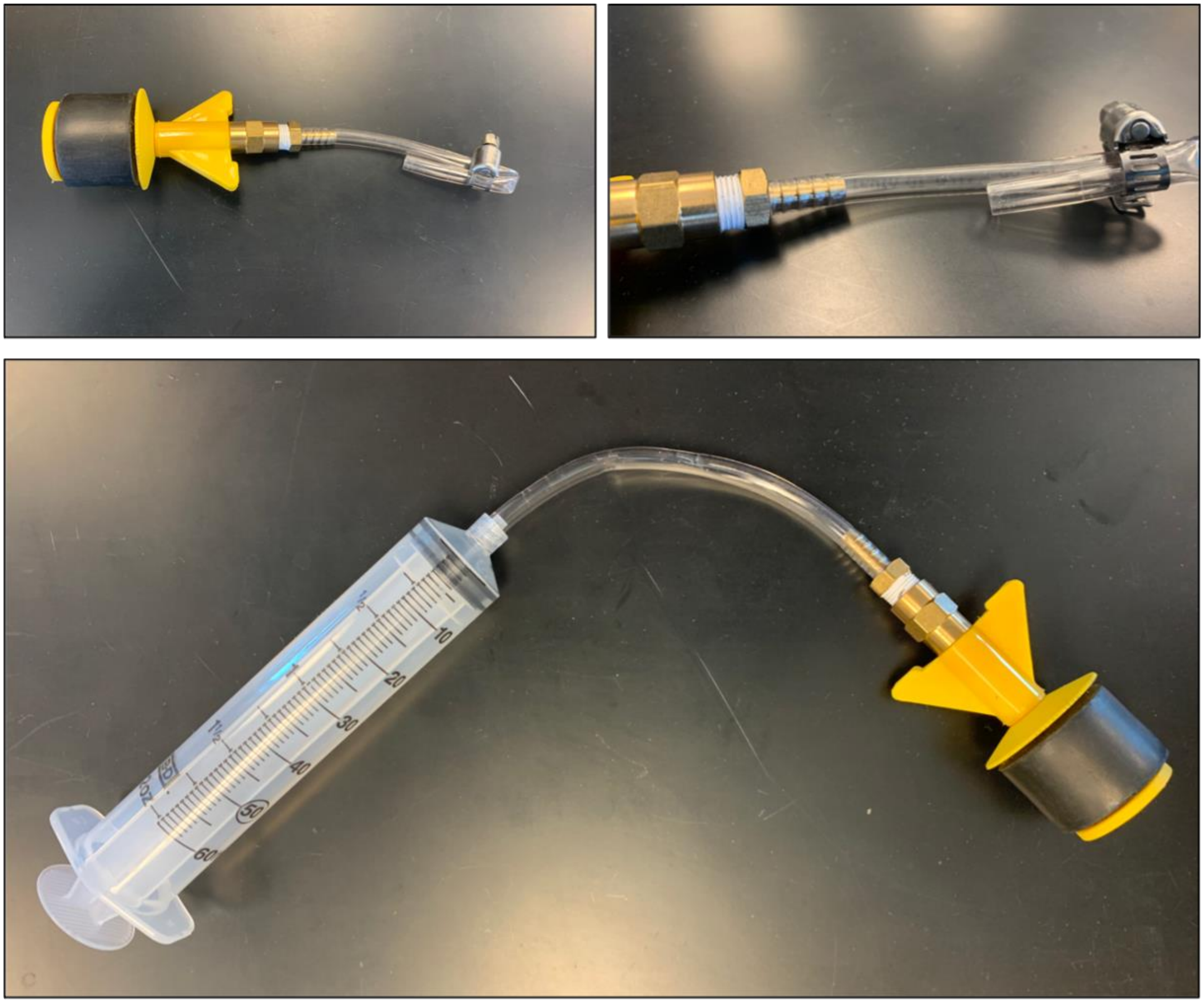

Figure S4. Custom packer used to seal boreholes with leaking fluid. The black rubber end is inserted into the borehole and then compression-expanded with the plastic wing-nut to trap fluid. The plastic tubing is crimped to prevent any fluid from flowing out. The tubing can then be attached to a syringe in order to draw fluid out of the borehole for biological or geochemical sampling purposes.

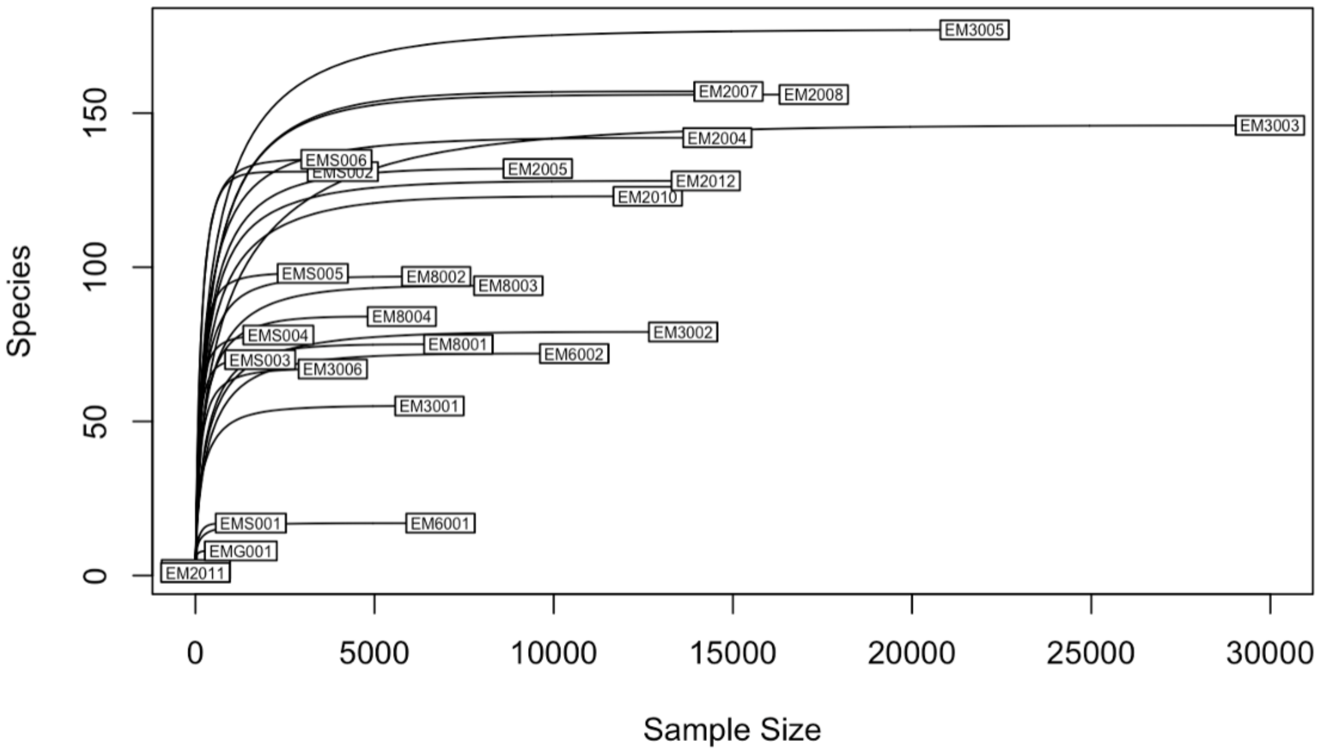

Figure S5. Rarefaction curves of samples collected from borehole and soil samples. Each curve is representative of an individual sample with the diversity mean species richness plotted on the y-axis and number of sequences on the x-axis. A rarefaction value of 5,761 sequences for the dataset used in this study to observe all borehole fluid samples, a rarefaction value of 1,270 was used to observe borehole fluids and other samples for ordination.

Table S1. Full geochemistry of borehole fluids from Edgar Experimental Mine
